# Jewish Hospitals in 20th Century Amsterdam: A Tale of Growth, Change, and Decline

**DOI:** 10.5041/RMMJ.10512

**Published:** 2023-10-29

**Authors:** Jack Y. Vanderhoek

**Affiliations:** Department of Biochemistry and Molecular Medicine, School of Medicine and Health Sciences, The George Washington University, Washington, DC, USA

**Keywords:** Amsterdam, hospital, Jewish, 20th century, the Netherlands

## Abstract

Major improvements in medical diagnostics and treatments in Dutch hospital care during the second half of the 19th century led to a shift from a nearly exclusive focus on indigent patients to an increasing proportion of hospital beds dedicated to paying middle-class patients. To accommodate this change, three private non-sectarian hospitals for middle-class patients were established in Amsterdam between 1857 and 1902. However, the two Jewish hospitals in the Dutch capital, the Dutch Jewish Ashkenazi hospital (NIZ), and the Portuguese Jewish hospital (PIZ), initially established exclusively for poor Jews, were much slower to respond to the trend of increasing hospital care for the middle class. This study examines how these hospitals addressed the needs of both poor and middle-class patients in the first decades of the 20th century as well as the success of the Centrale Israelitische Ziekenverpleging (CIZ, Central Jewish hospital) that was established solely for middle-class Jewish patients. The report also investigates how, after the devastation of the Amsterdam Jewish community during WW2, the CIZ managed to remain and today is the only ritually observant Jewish hospital unit in the Netherlands.

## INTRODUCTION

Since Jews first settled in the Netherlands at the end of the 16th century, Amsterdam has always been their most important city, both numerically and culturally. Between 1869 and 1899, the Amsterdam Jewish population grew from 30,039 to 59,117, although the proportion of Jews in the total city population remained constant at 11%. Poverty among the Amsterdam Jews was always a problem. For example, in 1870, the number of Jews who received permanent financial assistance was around 13,000 (43%), but this decreased to about 6,500 (11%) at the end of the 19th century.^1^

During the last third of the 19th century, there was a change in the socio-economic characteristics of the Amsterdam Jewish community. This was primarily due to the 1867 discovery of diamonds in South Africa. Between 1870 and 1890, there was a 10-fold increase in the number of Amsterdam diamond workers (to 10,000), 60% of whom were Jews, which made diamonds Amsterdam’s most important industry.^2^ Along with increases in other professions,^3^ these occupational changes led to increases in the income level and status of most Amsterdam Jews.

Hospital care in Amsterdam in the 19th century consisted primarily of several municipal and confessional charitable institutions for the ailing indigent population. However, by the first decade of the 20th century, hospital care had undergone major improvements and began to attract paying middle-class customers. These changes included the use of anesthesia and aseptic procedures, which led to more successful surgeries, and the availability of better diagnostic evaluations using X-ray machines, as well as the chemical and bacteriological results from the clinical laboratory to facilitate medical treatments, in addition to better trained nurses. To implement (some of) these improvements, three private hospitals were founded, the *Prinsengrachtziekenhuis* (1857, Prinsengracht hospital) and the *Boerhaave* clinic (1902) for the upper middle-class, and the *Burgerziekenhuis* (1879, Citizens’ hospital) for the lower middle-class.^4^–^6^

In contrast, the primary focus of the two Jewish hospitals in Amsterdam during that time was poor patients (i.e. those whose care was paid for by public funds), and the hospital facilities for middle-class patients were, at best, an afterthought. The largest Jewish hospital was the *Nederlands Israelitisch Ziekenhuis* (NIZ; the Dutch Jewish Ashkenazi hospital) with 110 beds, and the much smaller hospital was the *Portugees Israelitisch Ziekenhuis* (PIZ; the Portuguese Jewish hospital) which treated an average of six indigent patients ‘daily’.^7^ Before 1900, the NIZ had only one room (with at most four beds) available for middle-class patients, and the PIZ occasionally had one bed open for such a patron.8(a),^9^ In the late 19th and early 20th centuries, it seems likely that these few beds for middle-class Jewish patients were due to increasing requests for such hospital care, especially if home care was inadequate.8(b) If no room in either infirmary was available, these well-off Jews could be admitted to either neutral or confessional hospitals in which they could obtain kosher food but no other Jewish ritual services. However, for many Jews, whether religiously observant or not, the lack of a Jewish environment in these institutions influenced their choice of hospital in which they wanted to be treated.

It is commonly accepted that the more affluent, including Jews, usually prefer to be among those in similar circumstances. As more middle-class Jews preferred to be treated in hospitals, there was increasing interest in establishing “appropriate” Jewish hospital accommodations for these middle-class patients (e.g. more personal nursing care and rooms with one or two beds instead of a multi-bed ward for poor patients). This study will examine how hospital care for middle-class Jews developed in Amsterdam, how it compared to publicly supported hospitalization for poor Jews, and how it was influenced by the post-war Jewish demographics.

## HOSPITAL CARE FOR POOR AND MIDDLE-CLASS JEWS IN THE NIZ (1900–1940)

**Figure 1:**
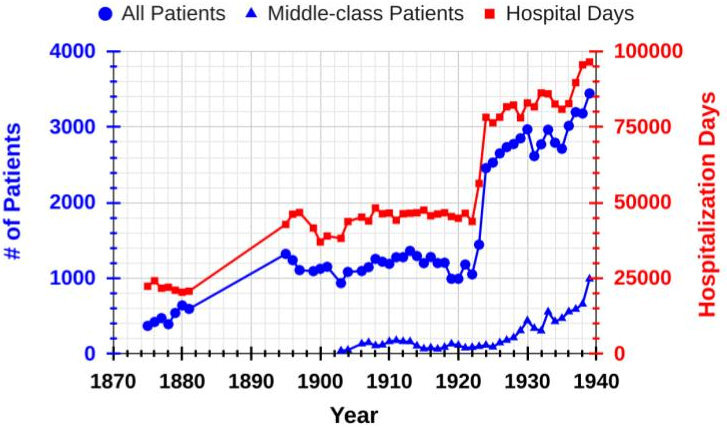
Patient Admission Data in the NIZ from 1875 to 1939. Note the different scales in Figure 1 compared to Figures 4 and 5.

**Figure 2:**
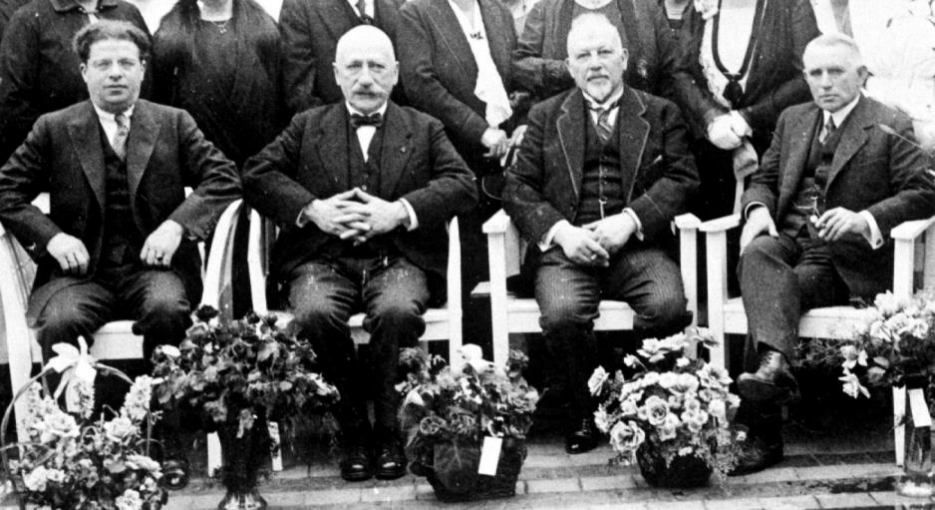
Photo of Dr A.S. Jacobson (1st from left), Dr S.J. Philips (2nd from left), and Dr M.H. Pimentel (1st from right) During Opening of the CIZ Solarium in 1920. Public Domain.

In 1883–1884, the *Nederlands Israelitisch Armbestuur* (NIA; Dutch Jewish Society for the Poor), the group of members of the Dutch Jewish Main [Ashkenazi] synagogue (NIHS) responsible for care of their communal poor, had replaced the previous Jewish hospital with a brand-new facility located on the *Nieuwe Keizersgracht* (#104–110) that contained 110 beds.^7^ The appointment of Dr A. Couvee as the NIZ medical director in 1886 resulted in several innovations such as the formal training of nurses, the use of an adjacent, newly designed home for nurses, and the establishment of some outpatient clinics as well as a small maternity ward.^10^ Although the NIZ was nearly exclusively for indigent Jews whose expenses were partly or wholly paid by the municipality, the data in Figure 1 show that the NIZ also admitted middle-class patients as early as 1903, although the number of these patients was much smaller than the total number of treated patients. (It is possible that the 1902 opening of the non-sectarian Boerhaave clinic might have influenced the NIZ’s decision to begin caring for paying middle-class patients.) By 1920, increased demand for patient beds, new innovations in medical care (e.g. surgery, maternity, and pediatrics11(a) that were not available in the 1884 building), the need for additional isolation rooms for patients with contagious diseases, and the realization that the hospital structure was no longer meeting modern sanitary and hygiene standards led to a decision to rebuild and expand the hospital. This was accomplished under the direction of the well-known and highly respected Dr S.J. Philips (Figure 2) who had been appointed board chairman of the NIA in 1914. The rebuilt and expanded hospital was opened on May 2, 1922. The ground floor contained the modernized kitchen, outpatient clinics, and a large waiting room. This floor as well as the first floor had wards for female patients, while the second floor was for male patients. All wards, whether they contained 8 (most) or 12 beds, had large balconies for open-air treatment, and this emphasis on fresh air was also fostered by a large roof-garden for convalescents. Surgical patients were housed in men’s, women’s, and pediatric wards. The top floor comprised the obstetrics department with separate wards for pregnant and post-birth women, new-born babies and a pediatric ward, the X-ray department, facilities for light and diathermy treatments, and the surgery division containing three operating rooms.11(b),^12^ All these changes doubled the patient capacity of this renovated hospital to 240 beds.

However, the problem of sufficient bed capacity persisted as bed occupancy in 1924 was already 90%.^10^ Hence in 1928, the NIA decided to again expand the NIZ, and a new wing was opened on July 1, 1931, which increased the bed capacity from 240 to 300 with four new wards (each for eight patients) on the ground and first floors. It also included new X-ray and laboratory facilities on the third floor.8(c) In addition, the outpatient service of the NIZ had become a very important component of the hospital’s medical care. In the 1930s, there were nine different outpatient clinics which ranged from the very busy surgical, dermatological, and obstetrics consultations to the less visited ear, nose, and throat (ENT), neurological, and pediatric specialties.8(d)

The data in Figure 1 show a substantial increase in the number of treated patients after the hospital expansion of 1922 (but not after the 1931 expansion). However, both expansions also helped accommodate more middle-class patients as shown in the steady upward trend until 1938 (the number of these patients in 1939 was unusually high), at which time 660 middle-class patients were treated, representing 20% of all patients in the NIZ.

## HOSPITAL CARE FOR POOR AND MIDDLE-CLASS JEWS IN THE PIZ (1900–1940)

The *Portugees Israelitisch Ziekenhuis* (PIZ) was the hospital arm of the Portuguese Jewish Society for the Poor of the Portuguese Jewish community.^7^ At the start of the 20th century, it was not surprising that, seven decades after its opening in 1834, the PIZ was in poor shape. Compared to the NIZ, the PIZ was much smaller. For example, in 1902, 130 patients were treated in the former whereas only 16 were nursed in the PIZ.13(a) Four years later, “the medical and nursing care was [considered] outstanding,” but the PIZ physical plant was in bad shape and no longer met the requirements of a modern hospital.8(e) Furthermore, admissions to the PIZ were down as physicians were instead recommending home care for their patients who preferred this over treatment at the PIZ or at other hospitals.11(c) Discussions within the *kerkenraad* (governing board) of the *Portugees Israelitische gemeente* (Portuguese Jewish community) led to the decision to build a new PIZ and Old Age Home for Women since both were historically part of the Portuguese Jewish Society for the Poor. A gift of *f* 100,000 (corresponding to 1.4 million euros today) from Mrs Eduard Teixeira Mattos for this new building was immensely helpful, and three land parcels on the *Plantage Franschelaan* (#8, 10, and 10a) in the center of Amsterdam were purchased for this purpose in 1914.8(f) On Monday, May 22, 1916 the new PIZ and Old Age Home for Women was opened. The hospital part of this new facility included (on the ground floor) a children’s ward with eight beds, a doctor’s consultation room, living quarters for the nursing director, and dining and social rooms for nurses. The first floor consisted of two large wards for 24 indigent patients, one for men and the other for women, and the top (third) floor housed two operating rooms, a diagnostic and therapeutic X-ray facility, a laboratory, and several nurses’ rooms. The whole second floor was reserved for 18 middle-class patients for whom eight rooms were available with either one, two, or four beds.^12^ In contrast to the 19th century PIZ, which only treated indigent Portuguese patients, the new PIZ was receptive to the new trend of hospital care for more well-to-do Jewish patients of this community and ensured that one-third of the available beds were for middle-class patients. No data were available on the specific number of middle-class patients treated in the PIZ. Interestingly, in 1927, it was reported that this hospital, perhaps due to its small size, suggestive of more personal attention, was more popular among Ashkenazi patients (464) than members of the Portuguese Jewish community (104) or non-Jews (52).8(g) It seems likely that similar results were observed in other years.

## THE CENTRALE ISRAELITISCH ZIEKENVERPLEGING (CIZ, THE CENTRAL JEWISH HOSPITAL) (1911–1940), A HOSPITAL FOR MIDDLE-CLASS JEWS

### Building of the CIZ Hospital, 1911–1916

On Friday, June 22, 1883, a notice appeared in the Dutch Jewish weekly *Nieuw Israelitisch weekblad* that a meeting would take place that evening to discuss the desirability and possibility of starting a hospital for middle-class Jews.8(b) Nothing seems to have come from this meeting, and several subsequent attempts also failed to elicit sufficient interest to build such a hospital. It was not until 1911 that things changed, which was primarily due to Dr Abraham S. Jacobson who was the driving force behind the ultimately successful founding of a private hospital for middle-class Jews in Amsterdam.

Abraham Simeon Jacobson (Figure 2) was born on October 17, 1879 into an Orthodox Jewish family in Amsterdam and had five siblings. He studied medicine in Amsterdam, was licensed in 1906, was a specialist in ENT medicine, and was associated with the NIZ. In 1907, he married Rosa Granaat who also became an ENT specialist. From 1912 to 1921, Dr Jacobson was a member of the executive committee of the NIA.^14^ It seemed that several of Dr Jacobson’s relatives had needed hospital care and had to be admitted to institutions that were able to provide kosher food but where the lack of a Jewish atmosphere hampered their ability to carry out certain religious rituals. In order to remedy this situation, he decided to start a private Jewish ritually observant hospital primarily for “the middle-class and better-off Jewish circles.”9(a),11(d) After convening a preparatory organizational committee, he persuaded the leading Amsterdam Jewish physicians, Drs S.J. Philips and M.H. Pimentel (Figure 2), to become the board chairman and vice chairman, respectively, of the *Centrale Israelitische Ziekenverpleging Vereeniging* (CIZV; Central Jewish Hospital Association), whose goal, as stated in the organization’s by-laws, was to found and operate such a ritually observant hospital for all Dutch Jews (i.e. paying subscribers).^15^ The board consisted of seven (later nine) members, which had to include three physicians. Dr Jacobson chose to become the CIZV board secretary, and the first meeting of this organization was held on February 22, 1911.11(e) Discussions with both the NIZ and PIZ to combine forces with CIZV were unsuccessful.^16^ As a result of many subsequent CIZV committee meetings (e.g. committees for advertising, building, finance, lady supporters, etc.) and the great efforts by Dr Jacobson, other board members, and a group of dedicated volunteers, donations of nearly *f* 200,000 (including an annual subsidy from the *Nederlands Israelitisch Hoofdsynagoge* [NIHS, Dutch Jewish Main [Ashkenazi] synagogue]) from about 800 donors were collected in four years so that the first cornerstone was placed on April 21, 1915.8(h,i) Despite the outbreak of World War 1, the board decided to proceed with the construction of the hospital, and the official opening of the CIZ took place on July 4, 1916 in the presence of Amsterdam Mayor Tellegen, Chief Rabbis Onderwijzer, Vredenburg, Palache, and Pereira, and other notables.11(f) Interestingly, the opening of the privately supported CIZ took place less than two months after the launch of the new PIZ hospital.

### The CIZ Hospital, 1916–1940

**Figure 3:**
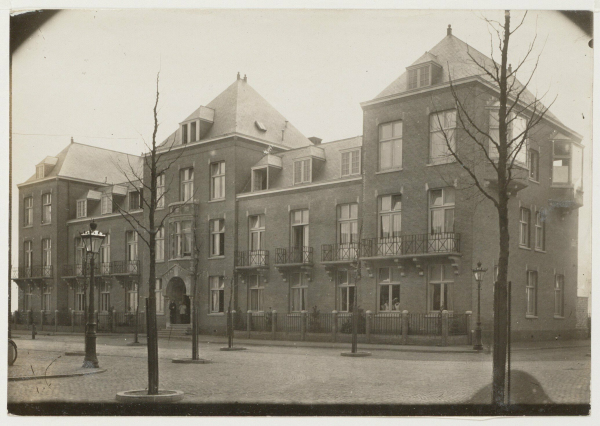
Photo of the CIZ (1920, Amsterdam Municipal Archives)

The nearly 2000-m^2^ property was located at 92 *Jacob Obrechtstraat* at the corner of the *Frans van Mierisstraat* and was purchased from the Amsterdam municipality. This location was situated in Amsterdam *Zuid* (South) which at that time was at the edge of the city and was one of the “better” neighborhoods. Objections by several CIZV members that this site was too far from the main center of the Jewish Amsterdam were overruled.^16^ The ground floor of the hospital building (Figure 3) housed third-class patients (four rooms, each with four beds) and contained waiting rooms, rooms for the director of nursing and for head nurses, dining and recreation rooms for the nurses, a board room, and the kitchen where kosher food was prepared. The first floor accommodated first- and second-class patients, nurses’ rooms, a nursery and children’s room, operating rooms, an X-ray facility, etc. The loft floor housed the staff. In addition, great care was taken to limit noise problems (to enhance patient convalescence) by the use of double-windows and rubber corridor carpets.8(i)^12^ In 1919, unique to Amsterdam, a roof-garden (solarium) with glass walls and roof was added where convalescents could enjoy the open air and sunlight and had a view of the garden and tennis courts. The hospital contained 52 patient beds (by 1924 it had increased to 56), and the per diem fee for inpatient hospital care ranged from *f* 20 for first-class patients to *f* 6 for third-class patients. Choice of physician was left up to the patient. Data from the first 10 years indicate that the proportion of third-class patients ranged from 38% to 51%, first-class patients from 4% to 15%, and second-class patients from 36% to 48%.^16^ Financial losses incurred from the third-class group were mostly offset by the income obtained from the first- and second-class patients. Initially, there were 13 in-house nurses, and this was usually augmented by other private nurses.

**Figure 4:**
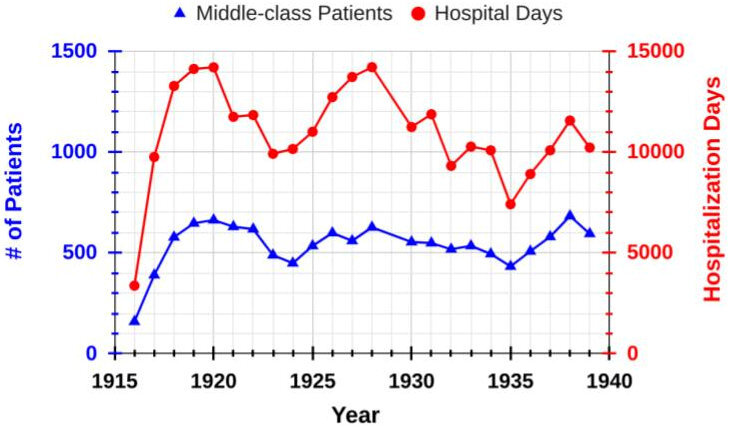
Patient Admission Data in the CIZ from 1916 to 1939.

For most of the pre-war existence of the CIZ, the number of annually admitted patients averaged about 560 and represented almost 11,300 hospital stay days, with a typical hospital stay of 25 days. Figure 4 shows the patient admission numbers from 1916 until 1939. It should be noted that Dr Jacobson, as the CIZV secretary, kept excellent patient hospitalization data for the CIZ. The annual number of newborns during this time period averaged about 43. During this time, the CIZ operated at a financial loss but managed to survive as a result of gifts, bequests, and an annual subsidy from the NIHS in Amsterdam. Several factors played a role in this situation. First, economic conditions were difficult during this period, even for the middle-class and the well-to-do. Second, there were consistent complaints regarding the relatively high per diem rate and other fees charged by the CIZ, although it was well known that other neutral (e.g. the Boerhaave clinic) or confessional hospitals charged comparable prices.8(j) Furthermore, there were instances when patients opted for a cheaper per diem patient class than their income status would have warranted.8(k) When the CIZV board introduced a second and cheaper per diem third-class rate in 1925 (which always operated at a loss), very few took advantage of this less expensive alternative.11(g) Third, it seems that many Dutch middle-class Jews did not take advantage of the CIZ but instead opted to be admitted to comparable non-Jewish hospitals mentioned above.8(l) Perhaps they preferred to be associated with non-Jewish middle-class patients and institutions rather than with the CIZ and its ritually observant milieu. It is interesting to note that at certain times, about 30%–40% of the CIZ patient population was not Jewish.11(g) Lastly, too few Jewish physicians seemed to recommend the CIZ as a preferred hospital, nor was there a lot of interest from Dutch Jews living outside Amsterdam.

Interestingly, in 1938 there were almost the same number (595) of middle-class patients cared for at the CIZ (Figure 4) as at the NIZ (660; Figure 1). This suggests that differences in either (1) the overall hospital emphasis (NIZ, primarily focused on poor individuals, versus the CIZ, a hospital for middle-class patients) or (2) the different locations of the NIZ (inner-city) and the more distant CIZ were not very important.

From 1911 until 1943, the CIZV leadership was quite stable, primarily due to the long service of Dr Philips as board chairman. Upon his death in 1934, he was succeeded by Dr Pimentel, as Dr Jacobson had declined this position due to health problems. In 1939, Dr Jacobson assumed the chairmanship of the CIZV.8(m)

## THE WAR YEARS AND THEREAFTER (1940–1970)

After the Germans invaded and occupied the Netherlands in May 1940, the situation for Dutch Jews worsened drastically. Many anti-Jewish laws were introduced. In February 1941, the German authorities established the *Joodse Raad* (Jewish Council), consisting of a group of Dutch Jewish leaders, which was responsible for enforcing Nazi orders as well as maintaining basic community services such as distributing food supplies, organizing housing, and providing medical care.^17^ To further isolate the Dutch Jews, as of May 1, 1941, the Nazi authorities prohibited Jewish doctors or Jewish hospitals from treating non-Jewish patients or employing non-Jewish personnel.^18^ In 1942, when the *Joodse Raad* indicated that neither sick patients nor hospital personnel would be removed from hospitals by the Nazis, there was a 10%–20% increase in the number of patients registered in all three Jewish hospitals and an upsurge in the numbers of Jewish hospital personnel as some Jews saw this as a means of guaranteeing themselves safety.^18^ At the start of 1943, there were 500 patients (normal capacity was 300) in the NIZ. However, as of January, 1943, the Nazis declared that sick patients or hospital personnel were no longer exempt from arrest or deportation.^18^ On August 13, 1943, the NIZ was closed and all remaining Jewish patients and hospital personnel were deported and murdered in the extermination camps in Poland (except for the few who managed to escape).^18^ By September 1943, all Jews had been removed and deported from the CIZ and PIZ.

When Dr Jacobson was elected as CIZV board chairman in 1939, he appeared to treat this position as akin to the hospital medical director and was more involved with the actual medical care of patients by the staff than he was with setting hospital policy and goals. Hence, it was not surprising that in March 1943, he had thanked the CIZ personnel who had remained on duty despite the increased deportations of Jewish patients.^18^ However, Dr Jacobson and his wife went into hiding in April 1943, because he was convinced that to stay was useless as the hospital management was about to be taken over by the Nazis. The Jacobsons were caught in August 1944 when the farm of the Dutch family Spriensma, with whom they were hiding, was raided by the Gestapo.^19^ They were deported to and murdered in Auschwitz on September 5, 1944. Farmer Reinder Spriensma was interrogated, imprisoned, and then transferred to Sachenhausen where he died on January 30, 1945.^20^ The Nazi objective to annihilate all Jews succeeded in murdering 75% of the 140,000 person-strong Dutch Jewish community.

In April/May 1943, the Nazis decided to use the CIZ and PIZ hospitals for mandatory sterilizations of intermarried couples. The Jewish partners of these couples and the assisting Jewish nurses were given the choice of either sterilization or deportation.^18^,^21^ When the sterilizations seemed imminent in May 1943, Dr Pimentel decided to go into hiding to avoid involvement in these inhuman procedures. The CIZ was closed for sterilizations on October 19, 1943, and the hospital was then transferred to the *Nederlandse Volksdienst* (Dutch Public Service) which operated it as a maternity facility for girls made pregnant by German soldiers.8(n) The PIZ sterilizations ended in June 1944.

By the end of the war, the NIZ and PIZ hospitals had been totally plundered. The NIZ was never reopened. After the liberation on May 5, 1945, the PIZ was initially used to house those returning from concentration camps who had no place to live.^22^ In view of the devastation of the Amsterdam Jewish community, the *kerkenraad* (governing board) of the Portuguese Jewish community eventually decided that it would be both impractical and financially disastrous to reopen the PIZ (especially since the CIZ had been designated to be the only remaining Jewish hospital in Amsterdam.^23^ The era of hospitals expressly supported by the Ashkenazi and Portuguese Jewish communities had come to an end.

The CIZ hospital building had also been severely damaged and most of its contents stolen. However, within weeks, it was partially restored under the leadership of returning board members Drs Pimentel and Groen and the director of nursing, Ms Duizend, in order to treat Jews who had returned from the concentration camps.^24^ It took several years to restore the hospital (including its X-ray and laboratory departments) to its previous condition, but the addition of a separate maternity ward helped defray the hospital’s operating costs.^24^,^25^ However, certain changes within the Jewish community as well as governmental regulations necessitated adjustments in the functioning of the CIZ.

**Figure 5:**
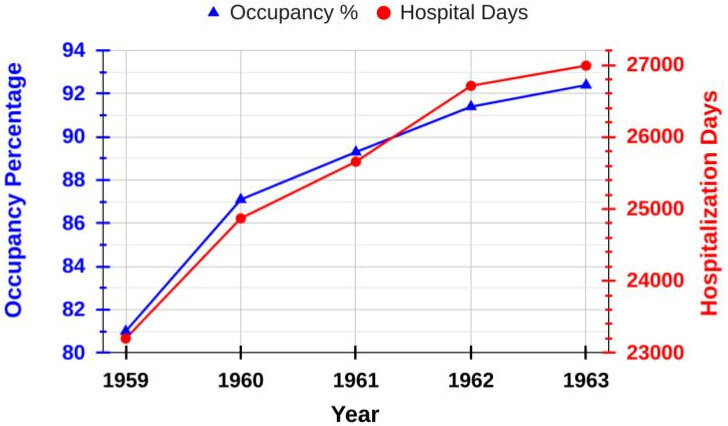
Patient Occupancy Percentages in the CIZ from 1959 to 1963.

Once the CIZ was selected to be the sole Jewish hospital,^24^ an agreement was made between the CIZV and the Jewish Social Work Foundation that allowed all Jewish patients, including the private-paying, the health-insured, and the indigent, to be admitted for treatment.8(o) Even chronic TB patients were welcomed.8(p) As before, patients were free to choose their doctor.^24^ In contrast to the pre-war situation when the CIZ had only a kosher kitchen as stipulated in its by-laws, the post-war hospital initially needed to maintain both a kosher as well as a non-kosher kitchen due to the lack of sufficient kosher food.^26^ After several years, sufficient kosher food had become available, but the CIZV leadership decided to continue the two-kitchen status quo, which led the chief rabbinate to withdraw its ritual authorization. It seemed that the majority of the CIZV board members were assimilated Jews who appeared to have little understanding or regard for the importance of rabbinical certification for a Jewish hospital for observant Jews, even though this was stipulated in the original CIZV by-laws. Consequently, those Jews who valued ritually supervised food were compelled to utilize non-Jewish hospitals (which provided kosher food) instead of the CIZ, a somewhat absurd situation. It was only after a legal battle between the CIZV and one of its members that an agreement was reached between the CIZV board and the rabbinate, whereby the latter agreed to provide ritual supervision as a result of changes in the admission procedures of Jewish patients.8(q),13(b) Less than 10 years after the war, more patient beds were needed (e.g. bed occupancy was 90% in 1957),^24^ and plans for expansion were implemented. On October 9, 1958, a new wing was opened which was made possible by the financial assistance of the Jewish Claims Conference, the Clara Foundation (which had operated a pre-war TB sanatorium for Jewish children), and other gifts. This new wing increased the patient capacity to 80 beds, and contained two large operating rooms, special surgical departments for outpatients, and additional amenities for nurses.8(r) Some patient data from 1959–1963 are shown in Figure 5. The left-side *y*-axis represents the total patient occupancy percentage, which appears more typically used during this time than the previously utilized “admitted patient numbers” (Figures 1 and 4). A second expansion increased the bed total to 100 which was accompanied by the addition of 16 baby bassinets.13(c) In the 1960s, the bed occupancy was “high to very high” (with occasional waiting lists), though this decreased in the first half of the 1970s to 81%–85%.^24^

Due to the many adverse changes experienced by the post-war Jewish community as well as transformations in modern hospital care, the original CIZV by-laws of 1911 were updated. In 1966, the original goal of the CIZV to provide Jewish ritually observant hospital care was reconfirmed and enlarged to include (1) clinics for outpatient care and (2) training of nurses and other medical personnel. In 1979, additional minor changes were made as a result of the merger with the *Tulp* hospital and the creation of a Jewish wing in the Amstelland Hospital.^15^

## THE MOVE OF THE CIZ HOSPITAL TO AMSTELVEEN (1970–PRESENT)

Since the original building was too small and lacked such modern necessities as intensive care and recovery room facilities as well as the latest advances in medical technology, the CIZV board in the late 1960s started preparations for constructing a new and more modern hospital in order to improve the hospital’s operating efficiency and to combine several of its temporary buildings under one roof. However, permission to do so was denied by the government primarily because its policy towards hospitals had changed. The new policy, as announced by the Ministry of Public Health, emphasized large regional hospitals with a capacity of 300–400 beds and required the closure of small local hospitals with 100–200 beds.8(s) In addition, hospital operating efficiency would be enhanced by limiting patient stays to approximately 1–1.5 weeks. Consequently, in 1972 the CIZV board started merger talks with the 165-bed *Nicolaas Tulp* hospital in Amstelveen, since the Public Health ministry had given provisional approval for a merger if the CIZ patient capacity would be decreased to 70. The closure of the 60-year-old CIZ hospital would signal the end of independent Jewish hospital presence in Amsterdam proper and that the center of Jewish hospital care would move to Amstelveen, a municipality south of Amsterdam but still within the Amsterdam metropolitan area. The choice of the *Tulp* hospital as merger partner was not very surprising for several reasons. The leadership of the *Tulp* hospital had supported an interdenominational approach in fostering cooperation between Protestant, Catholic, and Humanistic patients since its inception in 1965 and were definitely interested in the possible addition of Jewish patients.8(t) The proviso that these patients would want to maintain their Jewish identity in a separate part of the merged hospital was not considered a problem. The merger would also increase the hospital’s operating efficiency as well as the required patient capacity. Finally, the Jewish population of Amstelveen (and its neighboring Buitenveldert) was increasing and would welcome a medical center that would cater to Jewish patients.

Under the leadership of CIZV board chairs Drs E. Leisen and R.H. Levi, the merger negotiations were successful and led to the addition of three Jewish members to the nine-member board of the merged hospital, to be known as the *Ziekenhuis Amstelveen* (the Amstelveen Hospital), and the establishment of a separate CIZ wing in which the Jewish identity, ritual, and atmosphere would be upheld. The latter included: (1) a kosher kitchen, (2) surgical and medical services, (3) a recovery room, (4) no discharge on Shabbat or Jewish holidays, (5) a Jewish host(ess), and (6) a synagogue.8(u) Operating rooms and laboratory services would have a place in the main hospital building. On May 8, 1978, the new CIZ wing was inaugurated when the first Jewish patients were transferred from the CIZ hospital building on the *Jacob Obrechtstraat* and admitted to the CIZ wing of the Amstelveen Hospital (now known as the Amstelland Hospital).8(v)

Despite the presence of this Jewish wing, the number of Jewish patients taking advantage of this facility was well below its capacity of 70 beds. For example, the hospital admitted 280 Jewish patients out of the total of 2600 in 1986.8(x) The reasons for this are not difficult to surmise. First, those patients who want kosher food could get such food at most hospitals in the country. Hence staying in a hospital near their home might be preferable to traveling to Amstelveen. Second, some Jewish patients might not be interested in having a Jewish ritual atmosphere during their hospital stay. Third, patients might prefer to be treated by physicians who did not practice at the Amstelveen Hospital. Recently, the number of Jewish patients has increased, especially the Orthodox patients who seem to prefer the Jewish wing.8(y) It should be noted that in contrast to the patient hospitalization data of the prewar CIZ, the post-war archival data, especially after the 1978 fusion with the *Tulp* hospital, are either very scarce or non-existent. This might be due to the possibility that no one in the CIZV considered this a sufficiently important feature to be preserved.

In May 2023, the author visited the CIZ Jewish section of the Amstelland Hospital. Some things have obviously changed since the 1978 merger. Among these are different patient admission procedures and certain medical care practices which have resulted in less cohesiveness among Jewish patients. However, the Jewish atmosphere, activities, and kosher kitchen remain a strong and attractive feature of this hospital division.

This study reviews the history of Jewish hospital care for both indigent and middle-class patients in the three Amsterdam Jewish hospitals. The only Jewish hospital to survive the devastation of the Amsterdam Jewish community was the CIZ. After the war, the CIZ hospital treated all Jewish patients regardless of income. This hospital remained independent until 1978, when it merged with the *Nicolaas Tulp* hospital to become the only ritually observant Jewish hospital section (of the Amstelland Hospital) in the Netherlands. Although the Amsterdam Jewish community has supported the fulfillment of Dr Jacobson’s dream of a ritually observant hospital for over a hundred years, it is an open question as to whether this Jewish hospital unit will remain of sufficient importance to Dutch Jews that they will continue to support its existence in the future.

## Abbreviations

CIZ: Central Jewish hospital
NIZ: Dutch Jewish Ashkenazi hospital
PIZ: Portuguese Jewish hospital
